# Community succession and straw degradation characteristics using a microbial decomposer at low temperature

**DOI:** 10.1371/journal.pone.0270162

**Published:** 2022-07-08

**Authors:** Xin Zhang, Qinggeer Borjigin, Julin Gao, Xiaofang Yu, Bizhou Zhang, Shuping Hu, Shengcai Han, Ruizhi Liu, Sainan Zhang

**Affiliations:** 1 Agricultural College, Inner Mongolia Agricultural University, Hohhot, Inner Mongolia, China; 2 Key Laboratory of Crop Cultivation and Genetic Improvement in Inner Mongolia Autonomous Region, Hohhot, Inner Mongolia, China; 3 Special Crop Research Institute of the Academy of Agricultural and Livestock Sciences of Inner Mongolia, Hohhot, Inner Mongolia, China; 4 Vocational and Technical College, Inner Mongolia Agricultural University, Baotou, Inner Mongolia, China; 5 Horticulture and Plant Protection College, Inner Mongolia Agricultural University, Hohhot, Inner Mongolia, China; South China Agricultural University, CHINA

## Abstract

This study explored changes in the microbial community structure during straw degradation by a microbial decomposer, M44. The microbial community succession at different degradation periods was analyzed using MiSeq high-throughput sequencing. The results showed that 14 days after inoculation, the filter paper enzyme and endoglucanase activities increased to 2.55 U·mL^-1^ and 2.34 U·mL^-1^. The xylanase, laccase, and lignin peroxidase activities rose to 9.86 U·mL^-1^, 132.16 U·L^-1^, and 85.43 U·L^-1^ after 28 d, which was consistent with changes in the straw degradation rate. The degradation rates of straw, lignin, cellulose, and hemicellulose were 31.43%, 13.67%, 25.04%, and 21.69%, respectively, after 28 d of fermentation at 15°C. Proteobacteria, Firmicutes, and Bacteroidetes were the main bacterial species in samples at different degradation stages. The dominant genera included *Pseudomonas*, *Delftia*, and *Paenibacillus* during the initial stage (1 d, 7 d) and the mid-term stage (14 d). The key functional microbes during the late stage (21 d, 28 d) were *Rhizobium*, *Chryseobacterium*, *Sphingobacterium*, *Brevundimonas*, and *Devosia*. Changes in the bacterial consortium structure and straw degradation characteristics during different degradation periods were clarified to provide a theoretical basis for the rational utilization of microbial decomposer M44.

## Introduction

Crop residues are a renewable biomass resource, and approximately 5.1× 10^9^ tons of straw are produced each year globally [1,2]. However, open-field burning of crop straw seriously wastes resources and generates air pollution. Straw returning can increase the soil organic matter [3], which is a common post-harvest practice for disposing of these agricultural residues. The inefficiency of lignocellulose degradation in field conditions is the main bottleneck for its broader utilization in agricultural practice, and it also causes environmental problems [4]. In addition, prolonged low temperatures in autumn and winter in the frigid regions of northern China have restricted the decomposition of returned straw. It has been reported that the biodegradation of straw is an effective method to improve the efficiency of straw degradation and is more ecologically friendly and less toxic than physical and chemical treatment methods [5–7]. Accordingly, the addition of a straw decomposer adapted to the application environment seems a promising approach to improve the straw decomposition efficiency. Due to its complex structure (lignin, hemicellulose, cellulose, etc.), the efficient degradation of lignocellulose requires the coordination and interaction of various microorganisms [8]. In addition, microbial communities undergo succession at different straw degradation stages. Zheng et al. found that the species *Alcaligenes*, *Parabacteroides*, and *Sphingobacterium* became the dominant bacteria in a microbial consortium LTF-27 during culturing. The weight loss of rice straw amounted to 58.5% on day 20 [9]. Microbial consortium BMC-9, a biomass of *Clostridium*, *Bacillus*, and *Geobacillus*, reached the maximum biomass on day 9, as well as the highest xylanase activity (1.79 U·mL^-1^) and carboxymethyl cellulase activity (0.37 U·mL^-1^) [10]. Research has shown that there are specific interactions between the cultivation environment and microbial consortium during straw degradation. The microbial species and abundance of microbial consortiums significantly affect the straw degradation process [11,12]. Therefore, it is necessary to study functional microbial communities and their succession during straw degradation. However, there is still a research gap on how to properly use decomposers to disintegrate straw in the frigid regions of northern China.

In a previous study, a microbial consortium M44 was screened from dried dung from Inner Mongolia, China. M44 remained relatively stable during corn stover decomposition [13], and the dry powder decomposer was prepared by using cryogenic freeze-drying. However, the straw degradation characteristics and key microbial community dynamics of M44 during straw degradation were unclear. The data from this study improve our understanding of the synergistic mechanism of decomposer M44 and also provide a foundation for further biotechnological studies.

## Materials and methods

### Lignocellulolytic microbial decomposer

The microbial consortia M44 has been previously reported and was screened by restricted subculture and low-temperature domestication. The main microbial composition was *Pseudomonas*, *Flavobacteria*, *Azospirillum*, *Trichococcus*, *Acinetobacter*, and *Brevundimonas* [13]. The microbial decomposer (1.0×10^8^ CFU·g^-1^) was obtained by freeze-drying, in which the ratio of filler carrier (straw husk powder: soluble starch: and bran = 2: 1.5: 4.5) and the fermentation liquid broth was 4: 1.

Microbial decomposer M44 (2.0 g) was precisely weighed and added into 20 mL aseptic water containing 7–10 glass beads. This mixture was placed in a 15°C constant-temperature oscillator and fully oscillated at 200 r·min-1 for 5–10 min The prepared agent suspension (5% (v/v)) was inoculated into 40 mL Mandels medium with 1.0 g straw and then cultured at 15°C under static conditions and repeated three times. Samples were taken on the 1st, 7th, 14th, 21st, and 28th days to determine the degradation characteristics of straw and the microbial diversity of the microbial decomposer M44.

### Medium and culture conditions

Mandels medium (M medium) was composed of K_2_HPO_4_ (3.0 g), NaNO_3_ (3.0 g), CaCl_2_ (0.5 g), MgSO_4_·7H_2_O (0.5 g), Fe_2_SO_4_·7H_2_O (7.5 mg), MnSO_4_·H_2_O (2.5 mg), ZnSO_4_ (2.0 mg), CoCl_2_ (3.0 mg), and distilled water (1 L). M medium (40 mL) and 1.0 g straw were added to a 100-mL triangular flask for subsequent subculturing, and then the straw degradation ratio and enzyme activity were measured. Following this, the mixtures were sterilized at 121°C for 20 min and set aside. Harvested straw (the maize variety was Xianyu696) was taken from the experimental field of the corn center of Inner Mongolia Agricultural University (110°28’ E, 40°32’ N). The selected corn straw had no diseases or insect pests, a complete surface without serious damage, uniform size, and uniform thickness. It was washed and dried (60°C) and cut into small pieces of 2–3 cm for use.

### Enzyme activity and straw degradation rate measurement

The suspension of the microbial agent M44 was inoculated in the M medium, and 5 mL of the fermentation liquid and degraded straw was taken on the 1st, 7th, 14th, 21st, and 28th days. The fermentation liquid was centrifuged at 4°C and 5000 r·min^-1^ for 10 min. The filter paper activity (FPase), endonuclease 1,4-β-glucanase activity (Cx), and xylanase activity were determined by the DNS method [14]. The activity of laccase (Lac) was determined by the ABTS method, and the activity of lignin peroxidase (LiP) was determined by the resveratrol method [15]. Straw was taken out and washed with distilled water. Then, the corn straw degradation ratio was determined using the weight loss method. The lignin, cellulose, and hemicellulose contents were determined by a cellulose analyzer (ANKOM A200i). The degradation rate of lignocellulose was calculated in triplicate.

### High-throughput sequencing

The fermentation liquid of decomposer M44 was taken on the 1st, 7th, 14th, 21st, and 28th days after inoculation and centrifuged at 4°C for 10 min at 5000 r·min^-1^. The supernatant was discarded, and the precipitate remained. This was repeated several times to ensure complete precipitation. Under aseptic conditions, a bacterial genomic DNA extraction kit (China Tiangen Biochemical Technology Co., Ltd.) was used to extract genomic DNA from samples at different culture times, and 1% agarose gel was used for electrophoresis. PCR amplification was performed with the primers 338F (5’-ACTCCTACGGGAGGCAGCAG-3’) and 806R (5’-GGACTACHVGGGTWTCTAAT-3’) of standard bacteria 16S V3-V5 region, and the product was purified and quantified. An Illumina MiSeq sequencer was used to construct qualified libraries for sequencing, and sequencing was completed at Shanghai Meiji Biomedical Technology Co., Ltd.

### Statistical analysis

Operational taxonomic units (OTUs) with a 97% similarity cutoff were clustered using UPARSE (version 7.1, http://drive5.com/uparse/), and chimeric sequences were identified and removed. The taxonomy of each OTU representative sequence was analyzed using RDP Classifier (http://rdp.cme.msu.edu/) against the 16S rRNA database (Silva SSU128) with a confidence threshold of 0.7. Data of enzyme activities and straw degradation characteristics were analyzed by IBM SPSS Statistics 25.0 (IBM Inc., Armonk, NY, USA, https://www.ibm.com/cn-zh/analytics/spss-statistics-software), and R (V3.6.1) (https://www.r-project.org/)and Origin 2018(https://www.Originlab.com) was used to create figures.

## Results and discussion

### Straw and lignocellulose degradation rates of microbial decomposer M44

The straw and lignocellulose degradation rates increased upon extending the culture time and tended to be stable in the late stage (S1 Fig). The straw degradation rate increased the most at 1–7 days of culture. The straw degradation rate after culturing for 21 days was 30.51%, accounting for 97.07% of the total degradation rate. 31.43% of straw was degraded at the end of the culture. Hua et al. also showed that rice straw degraded rapidly at 0–9 days of culture and tended to be stable at the later stage [16]. Changes in the straw components’ degradation rate were generally consistent with the straw decomposition trend. Among them, the degradation rates of hemicelluloses and cellulose were significantly higher than those of lignin, and the maximum degradation rates were respectively 21.69% and 25.04% at the end of the culture. The degradation rate of lignin was the highest from day 14 to 21 and reached the highest value of 13.67% on day 28, which was significantly higher than the value at the early stage of culture.

Previous research on the screening of straw degradation using microbial consortia has mainly focused on normal or high-temperature conditions, but the in situ decomposition of returned corn straw in autumn at low temperatures is often poor. Previous studies have indicated that the degradation rate of straw was about 50% at 30–40°C [7,17]. In our study, the corn straw degradation rate using microbial decomposer M44 reached 30.51% at 15°C on day 21. Although this is lower than what is suitable for medium and high-temperature microbial consortia studied by other researchers, it can be adapted to corn straw returning in cold and arid regions.

### Enzyme activities of microbial decomposer M44

In the early stage of straw degradation by M44, the FPase and Cx activities increased rapidly and peaked on day 14 with 2.55 U·mL^-1^ and 2.34 U·mL^-1^, respectively, which were significantly higher than on the 1st, 21st, and 28th days (S2A Fig). The xylanase activity increased slowly after the 7th day, reaching 9.86 U·mL^-1^ at day 28 (S2B Fig). This was mainly because polysaccharides, amino acids, organic acids, proteins, and other soluble organic matters in straw were readily used by microorganisms in the early stage of degradation, and microorganisms secreted more cellulase and hemicellulase, which degraded straw. Therefore, the FPase, Cx, and xylanase activity increased rapidly in the early stage of culture. After culturing for 21 days, the Lac and LiP enzyme activity increased and reached 134.43 U·L^-1^ and 86.88 U·L^-1^, and then remained stable until day 28 (S2C Fig). Upon extending the degradation time, the easily-degradable components of straw gradually decreased, and highly refractory substances (such as lignin, waxy, and tannin) gradually increased, so the lignin activity increased rapidly in the later stage of culture. This result is consistent with changes in the straw lignocellulose degradation rate.

### Alpha diversity of microbial decomposer M44

Alpha diversity analysis was performed on the microbial communities of samples at different degradation stages (S3 Fig). The Ace index fluctuated slightly and reached the highest of 127.82 on day 21. The Shannon and Sobs indices increased from 1.29 and 58.67 on day 1 to 2.72 and 109.33 on day 28. The Simpson index decreased significantly from 0.41 on day 1 to 0.16 at the end of the culture. The alpha diversity indices showed no significant differences between days 21 and 28 of culturing, indicating that the diversity and richness of the microbial composition were most stable at the later stage of degradation.

### Beta diversity of microbial decomposer M44

Principal coordinate analysis (PCoA) based on weighted UniFrac distance was conducted for bacterial communities of samples at different culture periods (S4 Fig). There was a certain distance between each sample, indicating that there were differences in the species composition, relative abundance, and phylogenetic relationship of compound bacteria in different culture periods. To further clarify the differences, we performed ANOSIM and PERMANOVA analyses at the OTU level based on the Bray-Curtis distance algorithm. The results showed remarkable differences between samples at different culture stages (*p* < 0.05; N = 999 permutations).

PCoA showed that 21 and 28-day treatments clustered together, and there was no significant difference between the alpha diversity indices, indicating that the species composition abundance and structure were similar in the later stage of culture.

### Microbial composition dynamics using microbial decomposer M44

At the phylum level (others < 1%), the microbial decomposer M44 mainly contained Proteobacteria, Firmicutes, Bacteroidetes, and Actinobacteria. Most of the species related to the biomass cycle had relatively complete metabolic mechanisms, similar to the results of Wongwilaiwalin et al. [18], Zhu et al. [19], and Guo et al. [20]. In previous reports, Proteobacteria and Firmicutes were found in straw compost [21], rotten wood [22], and rumen [23]. These can secrete cellular laccase and peroxidase, which degrade the monoaryl, bisaryl, and phenolic intermediates of lignin and sulfate lignin [24–26]. Many studies have documented that Proteobacteria, Firmicutes, and Bacteroidetes are the main species involved in cellulose hydrolysis [27,28]. Among them, Proteobacteria accounted for the highest proportion at different culture stages and reached the maximum of 92.89% culture on day 14. Contrary to Proteobacteria, the abundance of Firmicutes decreased from 11.33% on day 1 to 4.80% on day 14 and then increased to 7.18% at the end of the culture. The richness of Bacteroidetes increased significantly from 0.17% on day 1 of culture to 10.24% at the end of the culture. There was a significant difference between samples at different culture stages (*p* < 0.05). There were no significant changes in Actinobacteria throughout the culture process (S5 Fig).

At the genus level (others < 1%), there were significant differences in the abundance of dominant genera at different culture periods (*p* < 0.05) (S6 Fig). *Pseudomonas* was the dominant bacterium in the samples at different culture stages, accounting for 55.25% and 54.82% of the total bacteria on days 7 and 14. *Pseudomonas* possesses a high capacity to utilize and degrade petroleum hydrocarbons and is classified as a hydrocarbonoclastic microorganism [29,30]. Guo et al. [31] and Wang et al. [32] detected *Pseudomonas* in the composite microbial XDC-2 and LDC, which efficiently degraded lignin and aromatic compounds. The relative abundance of *Enterococcus* was the highest on culture day 1 (10.44%), which was much higher than in other culture periods. *Delftia* and *Ochrobactrum* accounted for the highest proportions on day 14, which were 5.83% and 1.26%, respectively. In addition, the relative abundance of *Kaistia*, *Novosphingobium*, and *Trichococcus* were the highest on day 21, with 3.58%, 1.90%, and 3.89%, respectively, which were remarkably higher than that on days 1, 7, and 14. It has been reported that *Trichococcus* produces lactic acid, formic acid, and ethanol and participates in the decomposition of organic matter at temperatures as low as -5°C [33,34]. The relative abundance of *Chryseobacterium*, *Sphingobacterium*, and *Rhizorhapis* was 2.90%, 3.09%, and 2.43%, respectively, on day 28, which was remarkably higher than that in the early stage of degradation. Among them, *Chryseobacterium* has been found to produce cellulase and protease, which degrade cell walls [35,36]; *Sphingobacterium* can utilize and degrade lignocellulosic material for growth and reproduction, resulting in efficient straw degradation, and are classified as hydrocarbonoclastic microorganisms [11]. During straw decomposition, *Paenibacillus* remained stable at 1.28–1.65%, and there was no significant difference among samples at different degradation stages. *Paenibacillus* sp. was reported to produce xylanases that specifically decompose xylans [37]. It has a high thermal stability and wide pH adaptability and has been widely used for industrial enzyme production [38].

Comprehensive analysis of straw degradation characteristics and species composition changes shows that in the initial stage of straw degradation (1, 7, and 14 days), the structure of straw was relatively complete, and there was sufficient oxygen. *Pseudomonas*, *Paenibacillusm*, and *Delftia* mainly used nutrients in the medium to proliferate in large numbers. This was accompanied by the secretion of degrading enzymes (FPase, Cx, and xylanase), while the cellulose and hemicelluloses in the system were decomposed into small-molecule acids and esters that provided nutrients for the growth of other microorganisms. It is speculated that the above bacteria may be functional microorganisms during cellulose and hemicellulose degradation by compound bacteria. At the last stage of cultivation (21–28 d), the contents of *Kaistia*, *Novosphingobium*, *Brevundimonas*, *Chryseobacterium* and *Sphingobacterium* continued to increase, and the cellulase activities (FPase and Cx) declined. The Lip, Lac activity, and lignin degradation ratio rapidly increased and stabilized at relatively high values. It is speculated that the above bacteria are functional microorganisms related to lignin degradation.

There were also other strains (*Kaistia* and *Rhizorhapis*) without lignocellulosic degradation activity in M44, and non-functional aerobic bacteria may play an important role in providing an anaerobic environment, as well as growth promotion and pH neutralization [39,40]. Overall, interactions among these bacteria in the microbial decomposer M44 might be responsible for the high straw degradation activity and stable maintenance of the community structure.

### Correlation between the microbial composition of M44 and straw degradation characteristics

Correlation analysis of the vital genera of M44 and straw degradation characteristics (S7 Fig) showed that the Cx and FPase activity were positively correlated with *Delftia*, *Pseudomonas*, *Paenibacillus*, and some bacteria that degraded straw or intermediate products in the colony. *Chryseobacterium*, *Brevundimonas*, *Rhizobium*, *Delftia*, and *Devosia* were positively correlated with the xylanase, Lac, and LiP activities. The above results showed that *Pseudomonas*, *Delftia* and *Paenibacillus* mainly participated in the degradation of straw in the early stage, which increased the activities of cellulase and hemicellulase, and degraded cellulose and hemicellulose components efficiently. *Rhizobium*, *Chryseobacterium*, *Sphingobacterium*, *Brevundimonas* and *Devosia* are mainly involved in the degradation of straw at the later stage, which improved the activities of laccase and lignin peroxidase and efficiently degraded lignin components.

## Conclusion

In nature, the complete degradation of straw is the result of interactions between many microorganisms. In this study, the microbial decomposer M44 was shown to secrete a variety of enzymes that synergistically degraded lignocellulose in straw. The cellulase activity was highest in the early stage of culture, while the lignin activity was highest in the late stage of culture. This was consistent with changes in the straw lignocellulose degradation rate. There were significant differences in the composition diversity and richness at different periods of straw degradation using M44, in which *Pseudomonas*, *Paenibacillus*, *Delftia*, *Brevundimonas*, *Devosia*, *Chryseobacterium*, *Rhizobium*, and *Sphingobacterium* were the dominant genera. Overall, this study provides a theoretical basis for further research on the degradation mechanism of the microbial decomposer, M44. It also provides theoretical guidance for straw biomass transformation and utilization.

## Supporting information

S1 Fig Degradation rate of decomposer M44 at different stages of cultivation. (A) Corn straw degradation rate; (B) Lignocellulose degradation rate. The same small letter means no statistically significant difference, and different small letters indicate statistically significant differences at *p* < 0.05. (Identical for all following figures.).
(ZIP)

S2 Fig Enzyme activity of decomposer M44 at different stages of cultivation. (A) Filter paper enzyme activity (FPase) and endonuclease 1,4-β-glucanase activity (Cx); (B) Xylanase activity; (C) Laccase activity (Lac), and lignin peroxidase activity (LiP).
(ZIP)

S3 Fig The alpha diversity indexes of decomposer M44 at different stages of cultivation. (A) Ace index; (B) Shannon index; (C) Simpson index; (D) Sobs index.
(ZIP)

S4 Fig Species variation PCoA during the straw degradation process by M44. (TIF)

S5 Fig Bacterial composition of M44 at different cultivation stages at the phylum level. The “others” represents bacteria whose relative abundance was less than 1.00% in each sample.
(TIF)

S6 Fig Circos cluster analysis of dominant genera (A) and different analyses at the genus level (B).
(ZIP)

S7 Fig Correlation analysis of dominant genera and straw degradation characteristics at different stages of cultivation of M44. Cx, Endonuclease 1,4-β-glucanase activity; FPase, Filter paper activity; Lac, Laccase activity; LiP, Lignin peroxidase activity; DCR, Cellulose degradation ratio; DHR, Hemicellulose degradation ratio; DLR, Lignin degradation ratio.
(TIF)

S1 Raw data (ZIP)
