# Supplementary figures and images for: Community succession and straw degradation characteristics using a microbial decomposer at low temperature
Source: PLoS One. 2022 Jul 8;17(7):e0270162. doi: 10.1371/journal.pone.0270162 (PMC9269364; doi:10.1371/journal.pone.0270162)

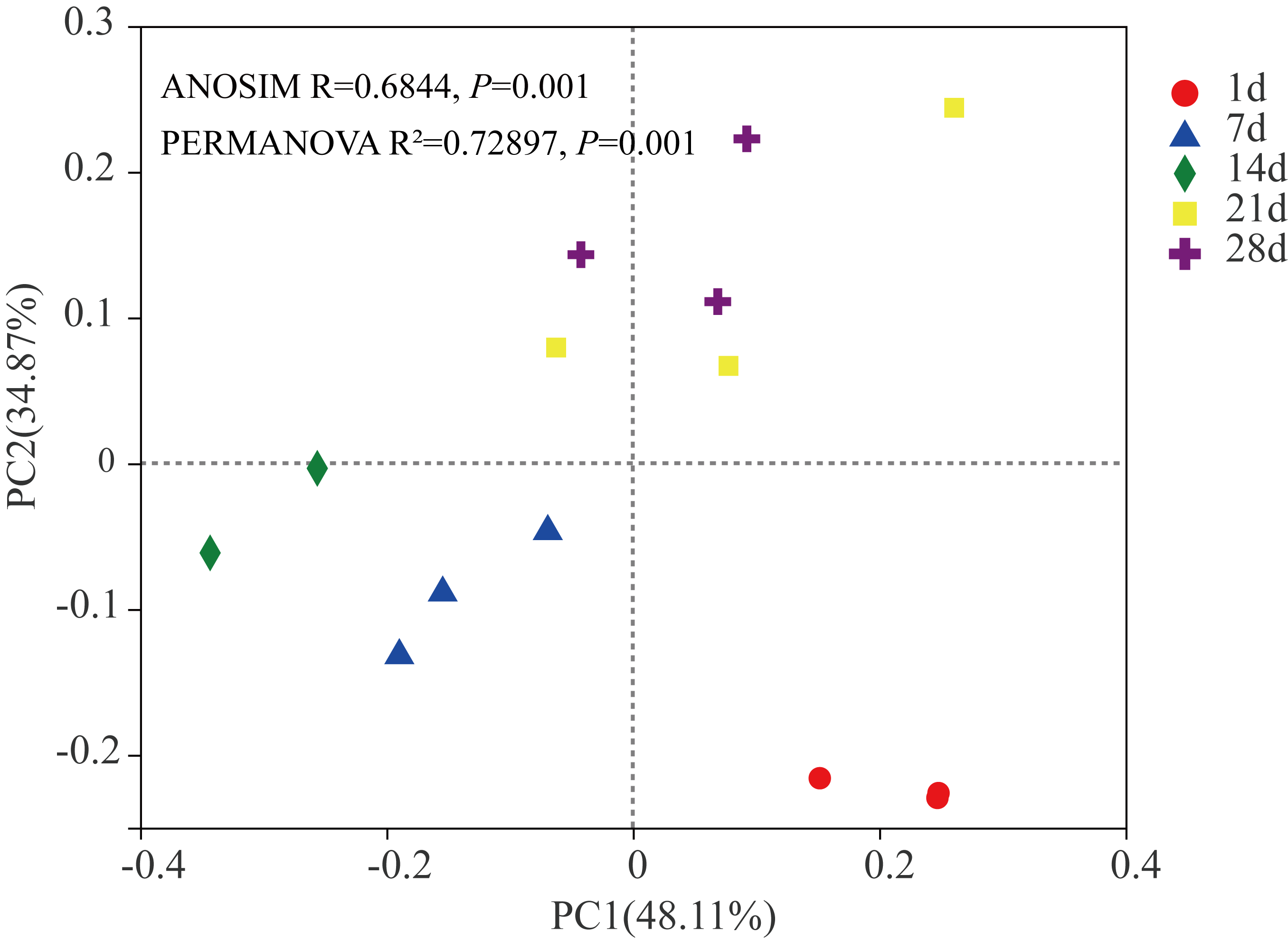

Supplement: S4 Fig — (TIF) [file pone.0270162.s004.tif]

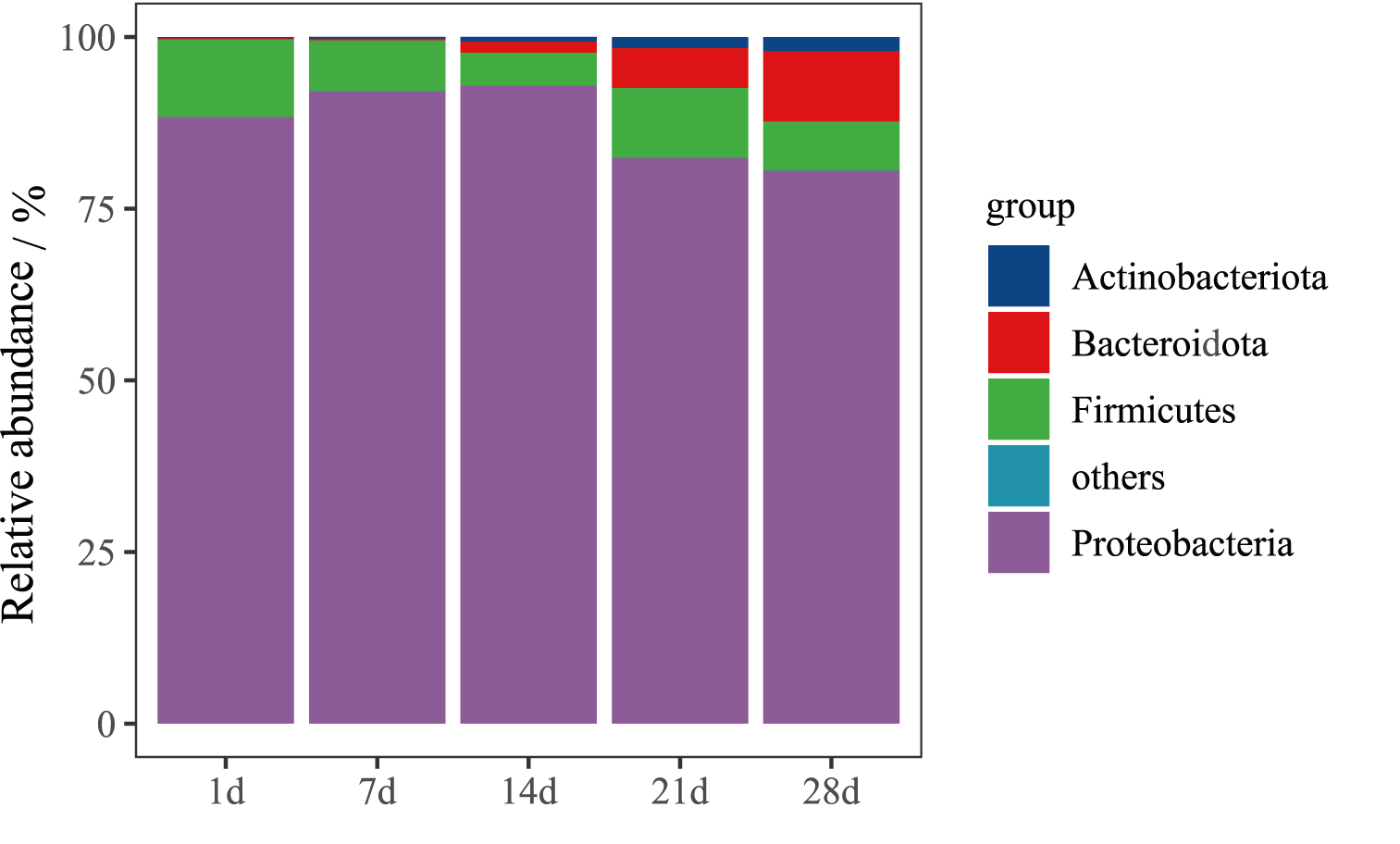

Supplement: S5 Fig — The “others” represents bacteria whose relative abundance was less than 1.00% in each sample. (TIF) [file pone.0270162.s005.tif]

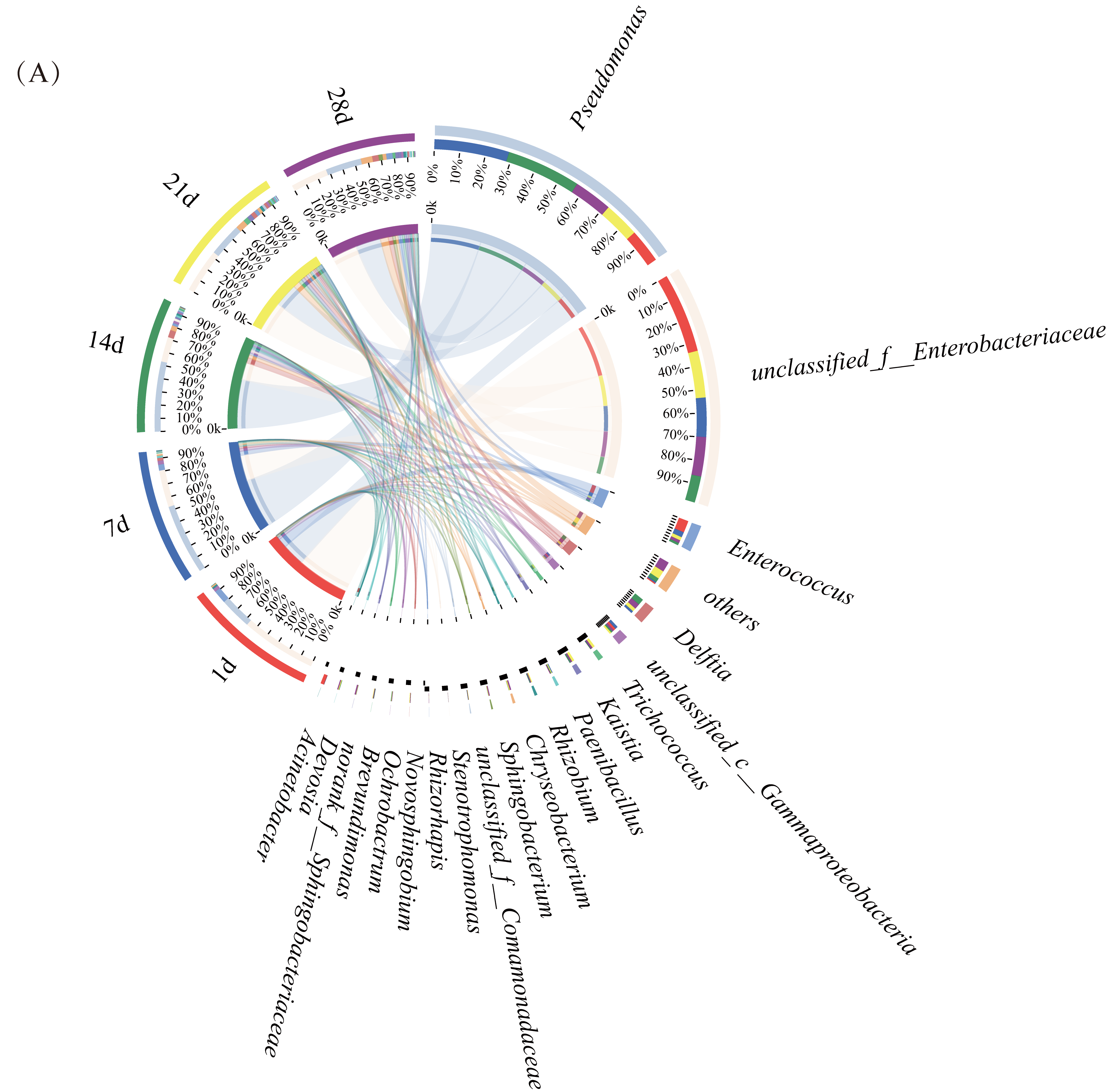

Supplement: S6 Fig — Circos cluster analysis of dominant genera (A) and different analyses at the genus level (B). (ZIP) [file pone.0270162.s006.zip › S6 Fig(A).tif]

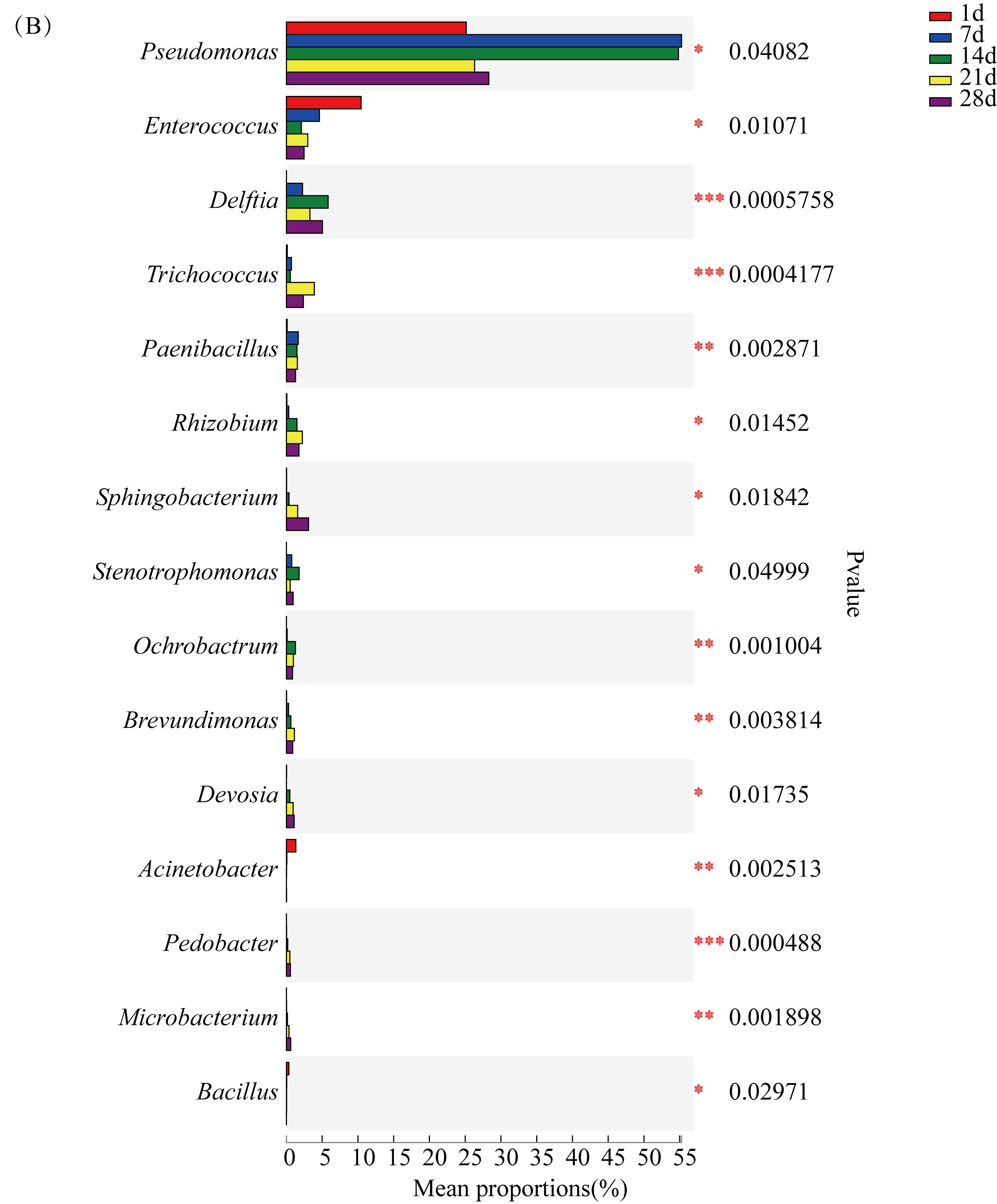

Supplement: S6 Fig — Circos cluster analysis of dominant genera (A) and different analyses at the genus level (B). (ZIP) [file pone.0270162.s006.zip › S6 Fig(B).tif]

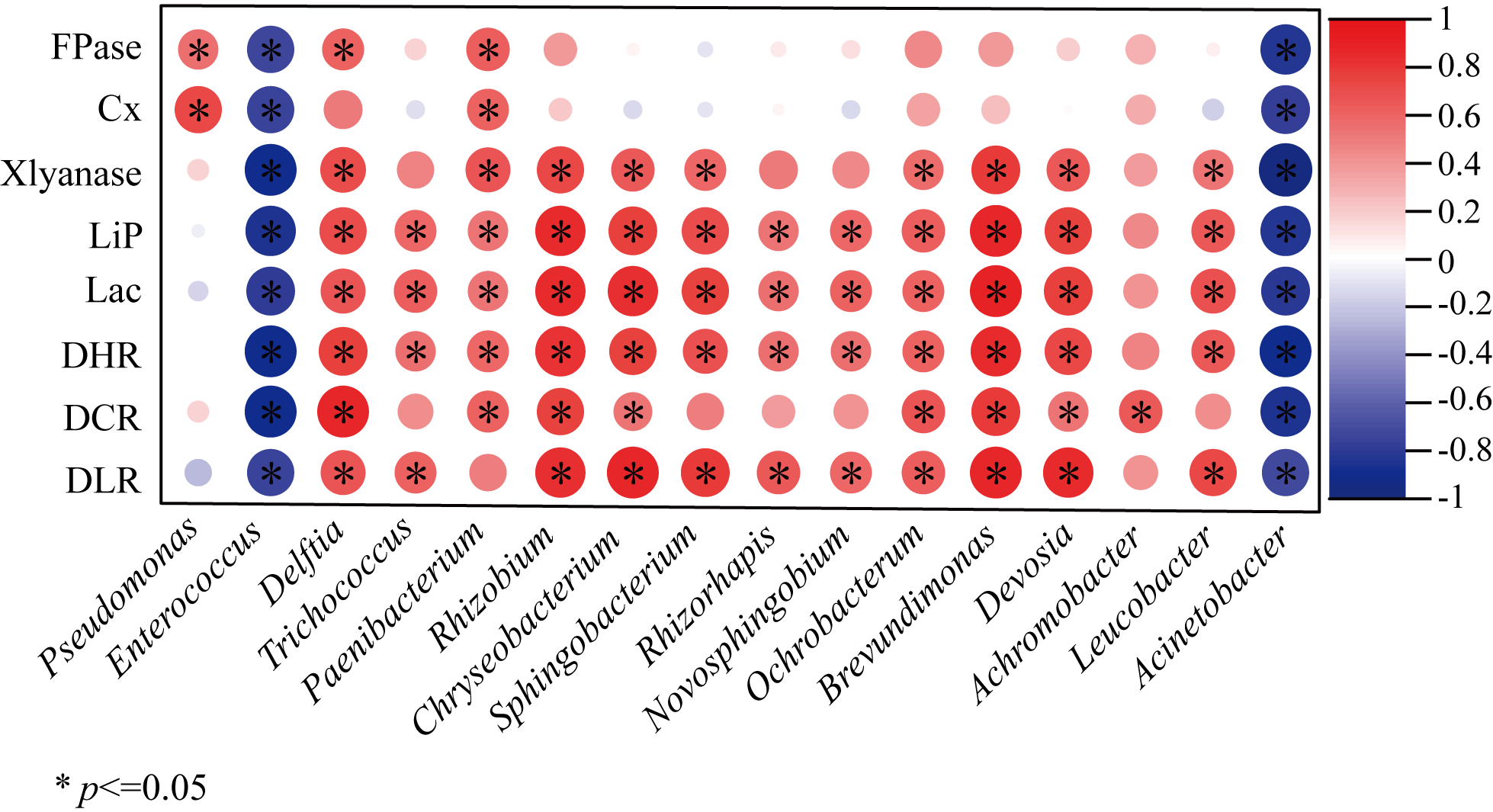

Supplement: S7 Fig — Cx, Endonuclease 1,4-β-glucanase activity; FPase, Filter paper activity; Lac, Laccase activity; LiP, Lignin peroxidase activity; DCR, Cellulose degradation ratio; DHR, Hemicellulose degradation ratio; DLR, Lignin degradation ratio. (TIF) [file pone.0270162.s007.tif]
